# Foramen of Winslow Hernias: A Case Series of a Rare Cause of Bowel Obstruction in Three Women

**DOI:** 10.7759/cureus.92729

**Published:** 2025-09-19

**Authors:** Hope M Cherian, Christia Lomas, Nima Khosravani, Monica Polcz, Jorge R Rabaza

**Affiliations:** 1 General Surgery, Herbert Wertheim College of Medicine, Miami, USA; 2 General Surgery, Florida International University, Herbert Wertheim College of Medicine, Miami, USA; 3 Gastrointestinal Surgery, University of South Florida Morsani College of Medicine, Tampa, USA; 4 Surgery, Baptist Health South Florida, Miami, USA

**Keywords:** epiploic foramen, foramen of winslow hernia, general surgery, internal hernia, intestinal obstruction

## Abstract

This case series describes three female patients between the ages of 51 and 66 who presented with epigastric pain with or without nausea, vomiting, and diarrhea. Each had minimal medical and surgical histories, most notably one with a congenital malrotation and prior cholecystectomy, one with an enlarged right lobe of the liver, and one with a prior hiatal hernia repair. CT results suggested a hernia through the foramen of Winslow (FWH) in each case, which was confirmed intraoperatively. Each patient underwent surgical treatment, two with a robotic approach and one with an open approach. One underwent a partial hemicolectomy and another a partial cecectomy for significant cecal dilation. All patients' postoperative courses were uncomplicated, and each recovered with no hernia recurrence or symptoms. These cases highlight the variability in presentation, imaging findings, and surgical decision-making associated with FWH.

## Introduction

Herniation through the foramen of Winslow (FWH) is one of the rarest types of internal abdominal hernias, accounting for approximately 0.08-0.1% of all hernias and up to 8% of all internal hernias [1]. Internal hernias cause only 0.2-0.9% of intestinal obstructions [2], making FWH extremely uncommon. First described by Blandin in 1834 [3], fewer than 300 cases have been reported [4].

The FWH, identified by Jacob Winslow in 1732 [5], is the natural communication between the greater and lesser sacs of the peritoneal cavity. Normally sealed by intra-abdominal pressure and visceral positions, it may become vulnerable to herniation with factors such as a mobile right colon, congenital malrotation, or prior surgical manipulation [6,7]. Symptoms are often non-specific, including vague or intermittent epigastric pain, nausea, or obstruction, making diagnosis challenging [8].

CT has emerged as a pivotal tool for diagnosis, allowing earlier recognition and management [9,10]. There is increasing use of laparoscopic and robotic repair over laparotomy [4]. No standard operative strategy exists, and management of incidentally discovered or spontaneously reduced FWH remains debated [6]. This study aims to present a series of three FWH cases managed at a single tertiary care center, highlighting variability in presentation, diagnostic imaging, and operative decision-making to contribute to the limited literature on this rare entity.

## Case presentation

Participants

Three female patients (ages 51, 52, and 66) presented between 2023 and 2024 with epigastric pain, nausea, or bowel-related symptoms. Two patients were White Non-Hispanic individuals, and one had no documented race/ethnicity. Each case was evaluated in a tertiary care setting, with preoperative imaging confirming or suggesting FWH.

Recruitment, pre-intervention optimization, and intervention

All patients underwent abdominal CT scans, medication reviews, and anesthetic risk assessments in accordance with institutional protocol. None required ICU stabilization. All patients underwent surgical intervention under general anesthesia without the use of mesh.

Case 1

**Figure 1:**
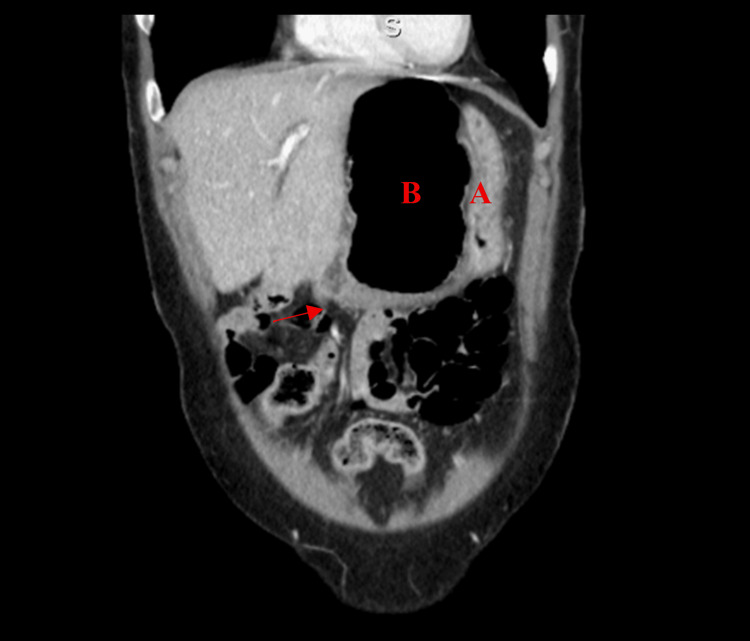
Anterior coronal view of Patient #1 Anterior coronal view of Patient #1. (A) Stomach and (B) the cecum pushing the stomach into the left-upper quadrant. Arrow: colon traveling through the foramen of Winslow.

**Figure 2:**
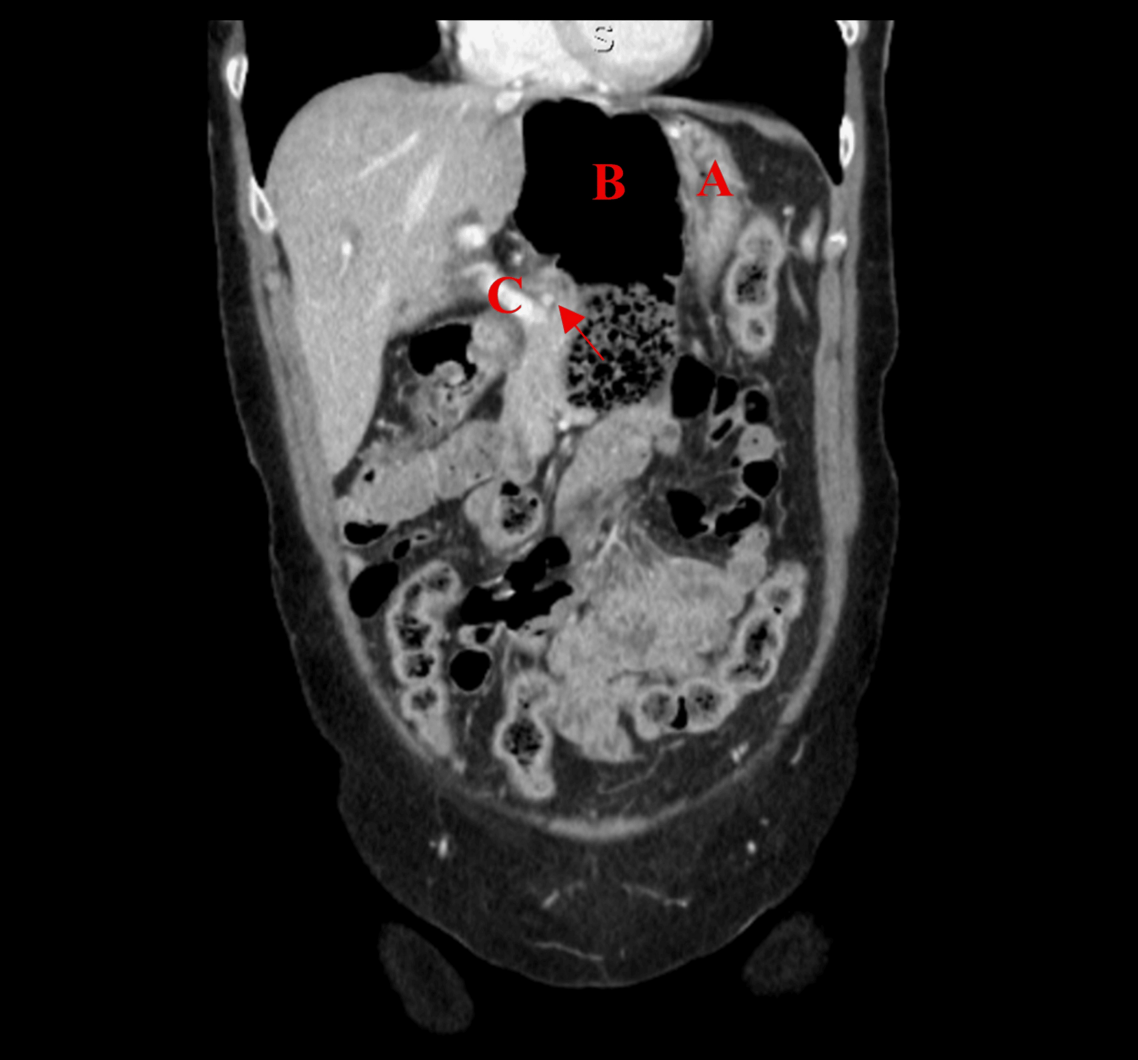
Posterior coronal view of Patient #1 (A) Stomach, (B) colon inside FWH, and (C) portal vein. Arrow: colon traveling through FWH. FWH: foramen of Winslow

**Figure 3:**
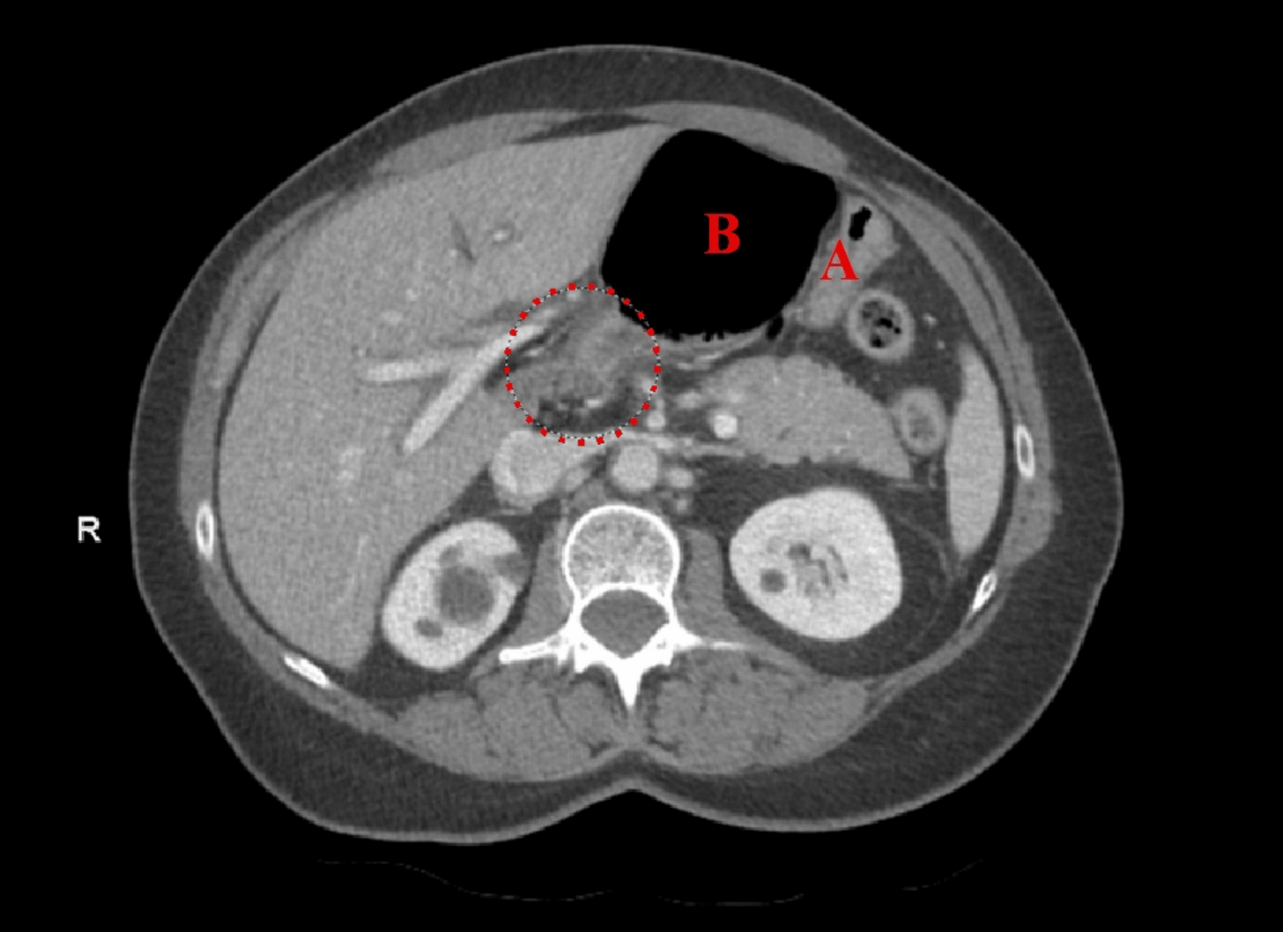
Axial view of Patient #1 (A) Stomach and (B) colon inside FWH. Dashed circle: colon traveling through FWH. FWH: foramen of Winslow

Patient #1 is a 62-year-old female with no chronic medical conditions but with a history of a laparoscopic cholecystectomy. The patient presented to the emergency department with sudden-onset, postprandial, colicky, epigastric abdominal pain with nausea. An abdominal CT was ordered with concern for bowel obstruction and showed suspicion of cecal volvulus (Figures 1-3). General surgery was consulted, and a nasogastric tube was placed to attempt bowel decompression.

**Figure 4:**
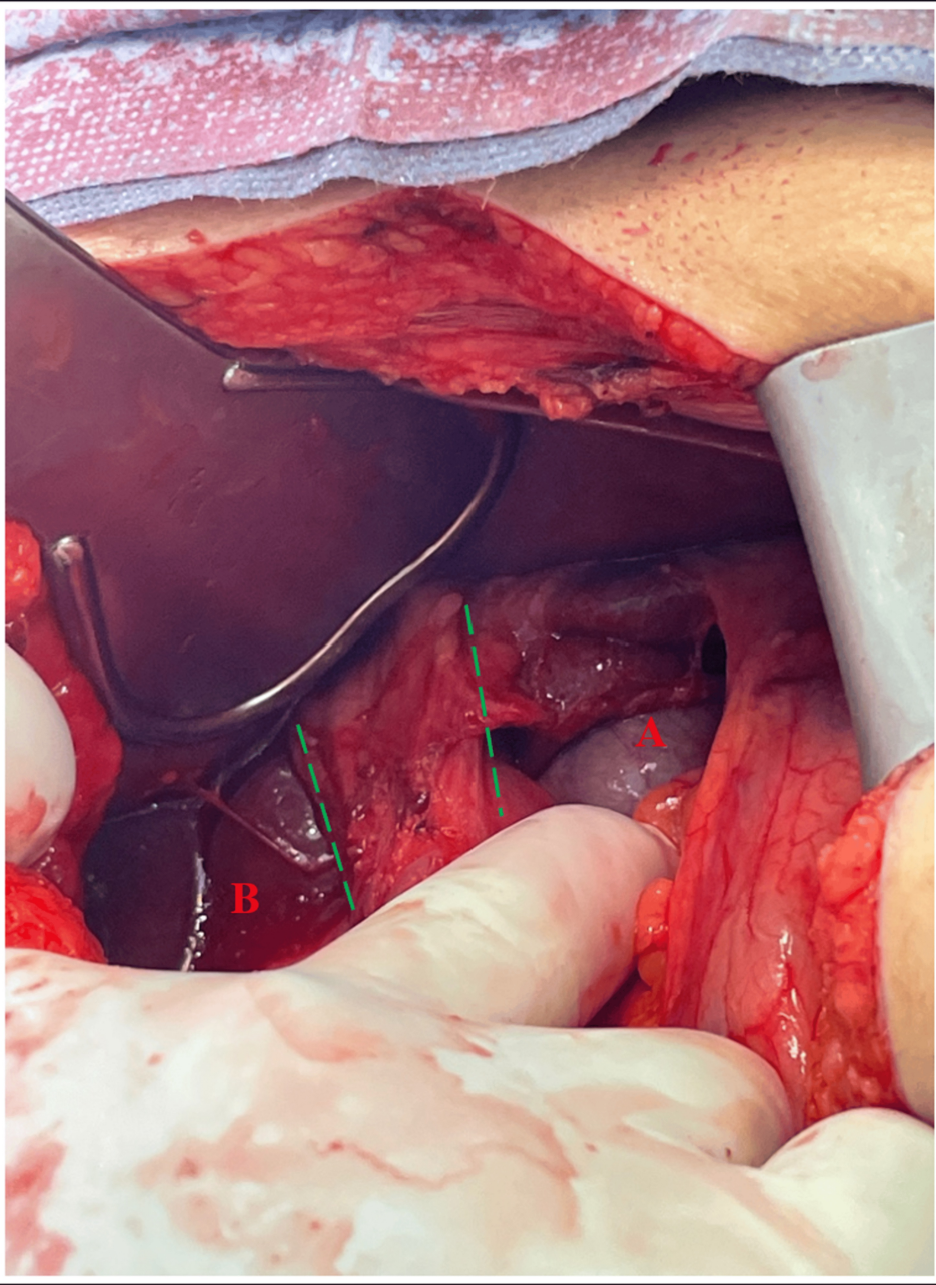
FWH after hernia reduction (A) Inferior vena cava and (B) caudate process of the liver. Dashed green lines: portal triad. FWH: foramen of Winslow

**Figure 5:**
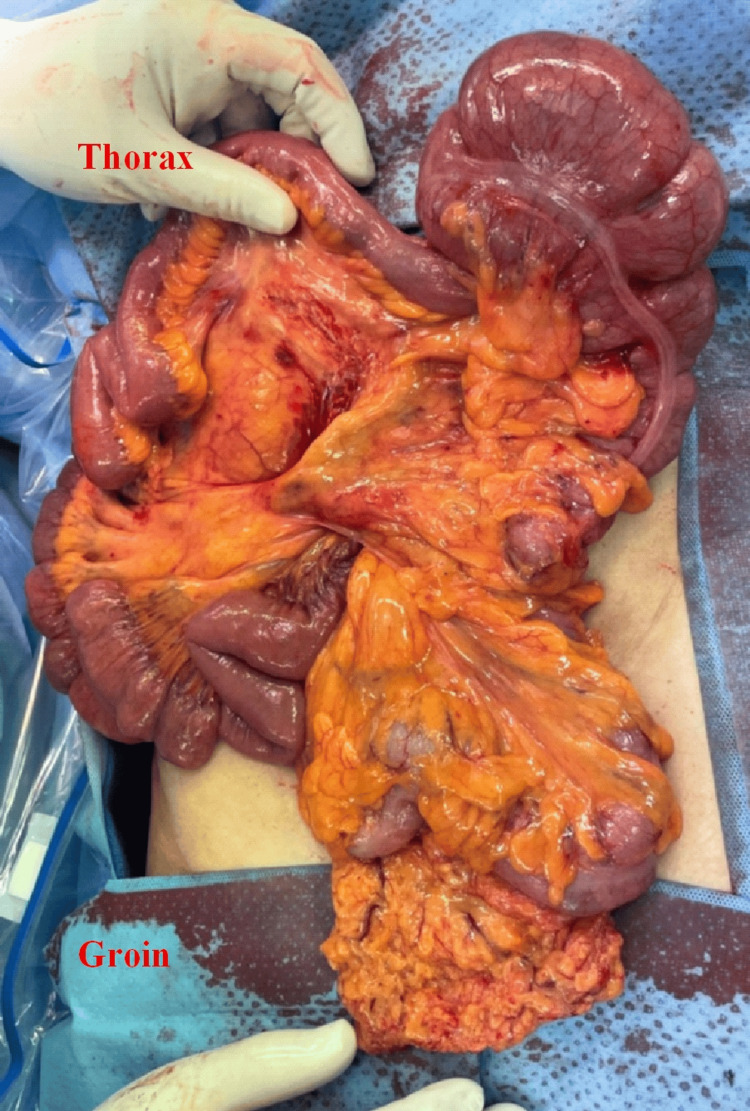
Orientation of abdominal organs confirming congenital malrotation Exposed small bowel, cecum and ascending colon, confirming the diagnosis of congenital malrotation.

Upon surgical exploration, the large bowel was noted to be herniating through a defect at FWH (Figure 4). Additionally, the large and small bowels were not in typical anatomic positioning (Figure 5), confirming congenital malrotation. There was laxity throughout the abdomen, and the bowels did not adhere along the abdominal sidewalls.

Upon examination of the herniated bowel, a right hemicolectomy was performed due to the risk of perforation from the resultant significant dilation of the cecum and ascending colon. A tongue of omentum was placed within the epiploic FWH to prevent herniation recurrence.

In summary, open laparotomy with reduction of the herniated cecum and ascending colon, right hemicolectomy, and placement of the omental tongue in the foramen were completed. The patient made a full recovery with no postoperative complications or recurrence of symptoms at the two-week follow-up.

Case 2

Patient #2 is a 51-year-old female with a past medical history of migraine disorder and Grover’s disease, without prior history of abdominal surgery. The patient presented to the emergency department with persistent, intermittent epigastric pain radiating to the middle back. An abdominal CT was completed outpatient the day prior due to severe abdominal pain, which showed the cecum within the lesser sac, associated with mesenteric swirling, and without signs of obstruction. The patient was advised to monitor symptoms. A repeat abdominal CT was ordered upon hospital presentation.

**Figure 6:**
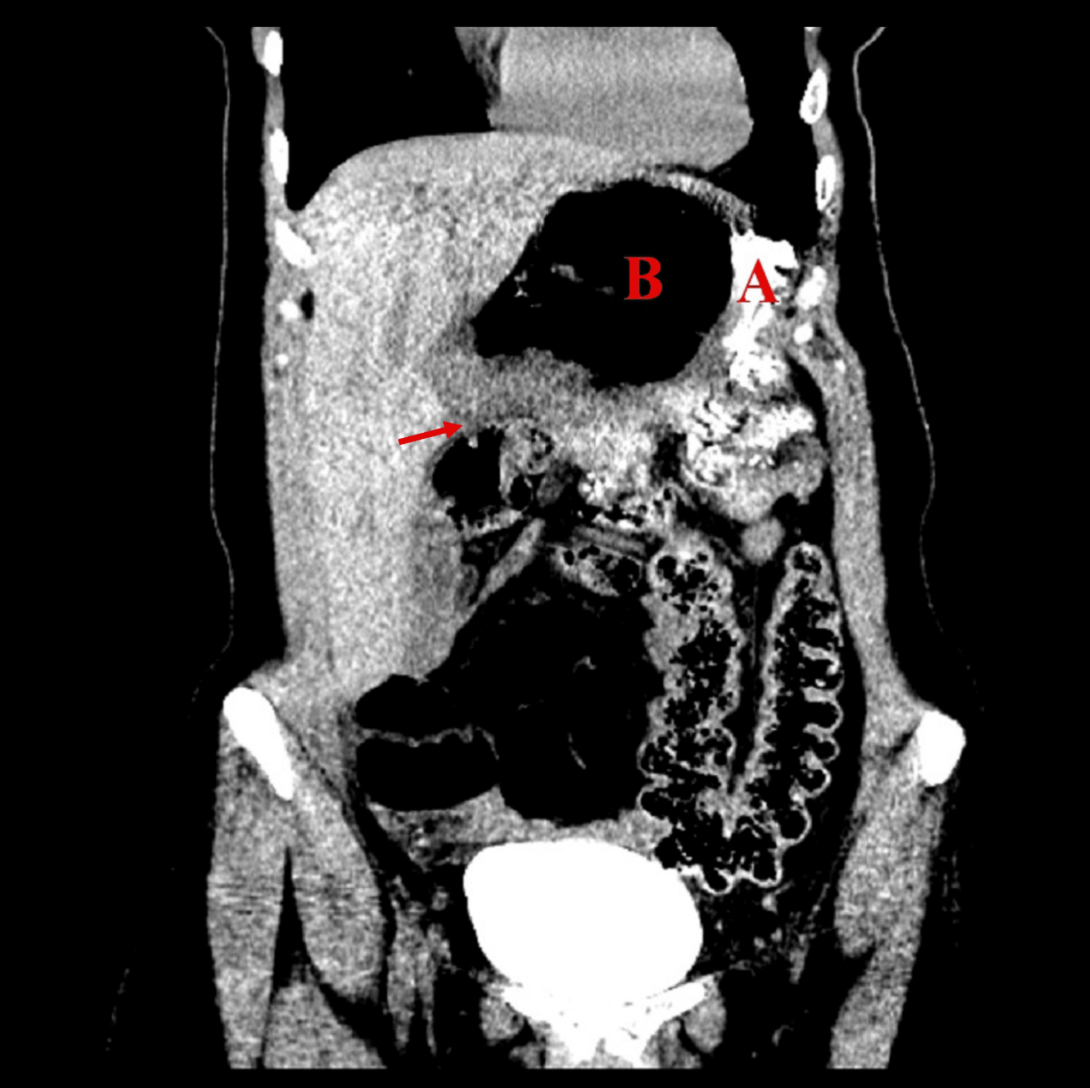
Anterior coronal view of Patient #2 (A) Stomach and (B) cecum pushing the stomach into the left upper quadrant. Arrow: colon traveling through FWH. FWH: foramen of Winslow

**Figure 7:**
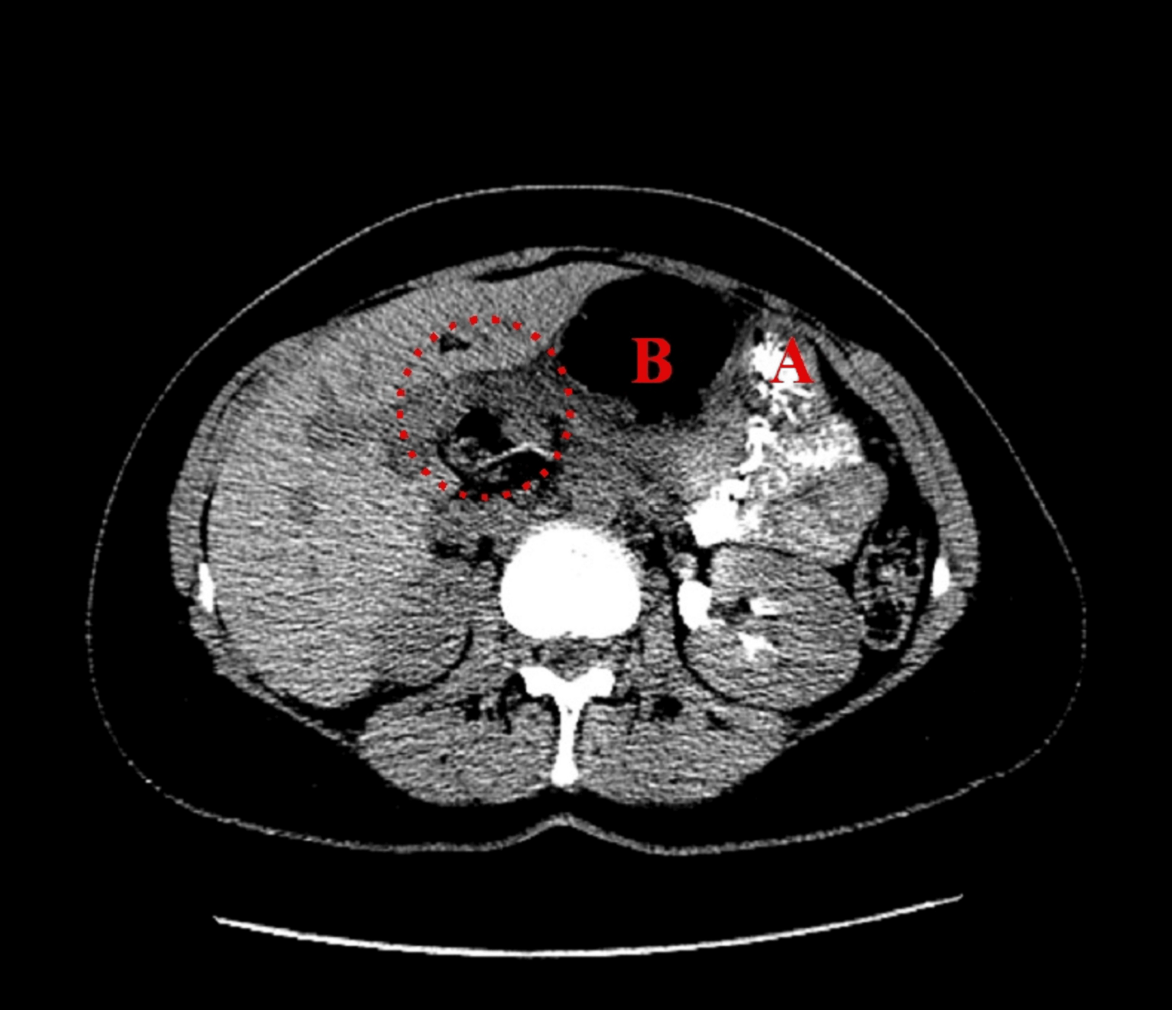
Axial view of Patient #2 (A) Stomach and (B) colon inside FWH. Dashed circle: colon traveling through FWH. FWH: foramen of Winslow

A new CT showed abnormal orientation of the bowel loops with the cecum and ileocecal valve located in the upper abdomen at the level of the lesser sac, with associated twisting of mesenteric vessels. This suggested the presence of an internal lesser sac hernia (Figures 6-7).

Soon thereafter, the patient experienced a quick resolution of pain, which raised suspicion of spontaneous reduction. Surgical versus nonsurgical management was discussed with the patient and her family. Using shared decision-making, the patient and surgical team agreed that operative repair was in the patient’s best interest. Both foramen closure and prophylactic ileocecectomy were discussed with the patient, who wished to proceed with foramen closure without bowel resection.

For robotic exploration, four 8 mm robotic ports were placed oriented horizontally at the level of the umbilicus. On inspection, the patient was noted to have a very elongated right lobe of the liver, extending into the pelvis. The gallbladder was elevated with a grasper, and a large defect at the FWH was visualized. There was adequate peritoneal tissue laterally along the hepatoduodenal ligament to reapproximate this defect safely to the retroperitoneum. A 2-0 STRATAFIX spiral suture was used to reapproximate small bites of the peritoneal tissue at the lateral aspect of the hepatoduodenal ligament to the retroperitoneum, closing the defect in running fashion. When locking the suture, small medial bites of a vascularized pedicle of omentum were incorporated to further reinforce and plug the area. The lateral aspect of this vascularized omental pedicle was fixated to the retroperitoneum with an interrupted 3-0 Vicryl suture, again taking small bites of the peritoneum only. Vistaseal was applied over this closure. The cecum appeared mobile but nonischemic. The patient made a full recovery with no postoperative complications or recurrence of symptoms at the two-week follow-up.

In summary, the patient underwent a successful robotic repair of FWH with omental buttress.

Case 3

**Figure 8:**
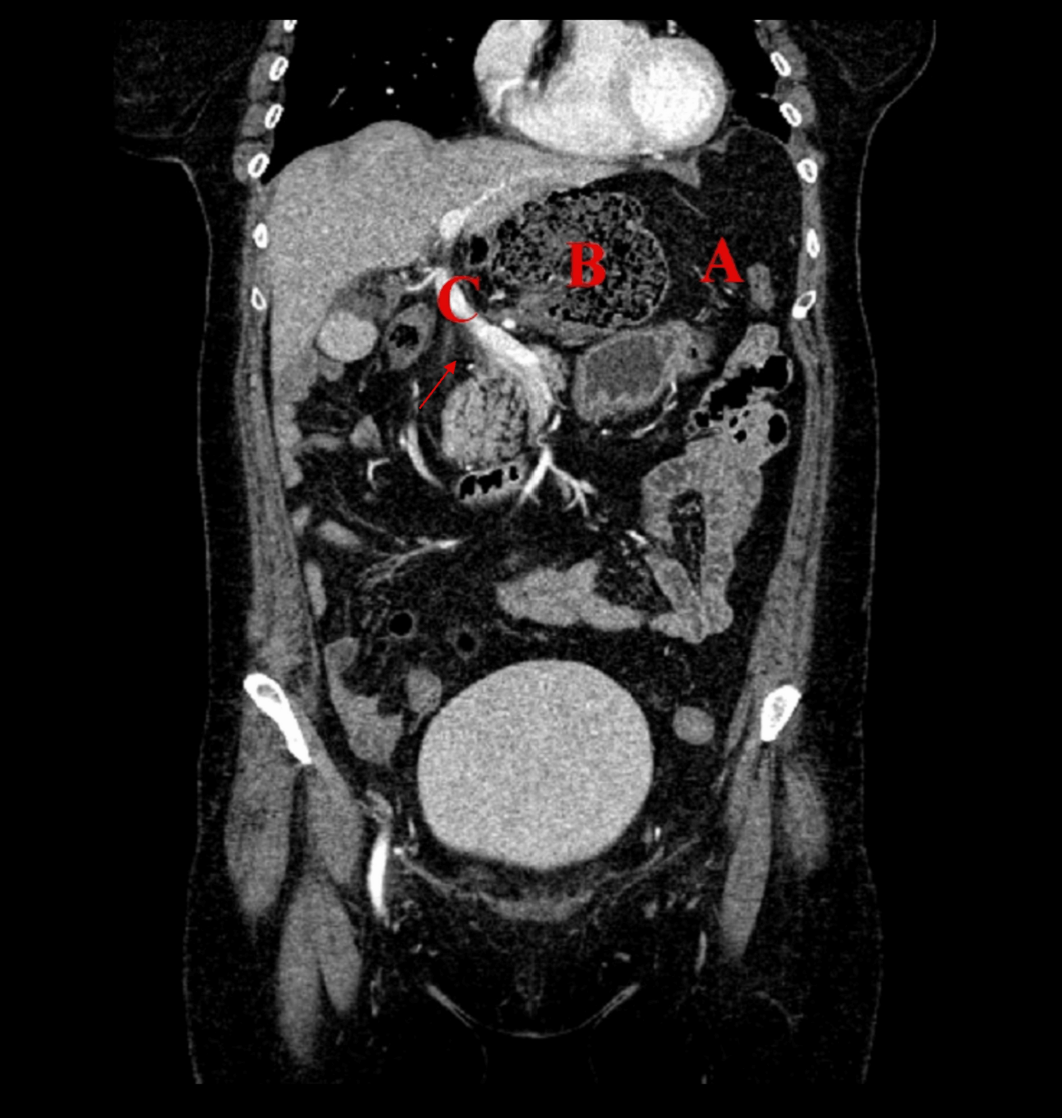
Anterior coronal view of Patient #3 (A) Stomach, (B) colon inside FWH, and (C) portal vein. Arrow: colon traveling through FWH. FWH: foramen of Winslow

**Figure 9:**
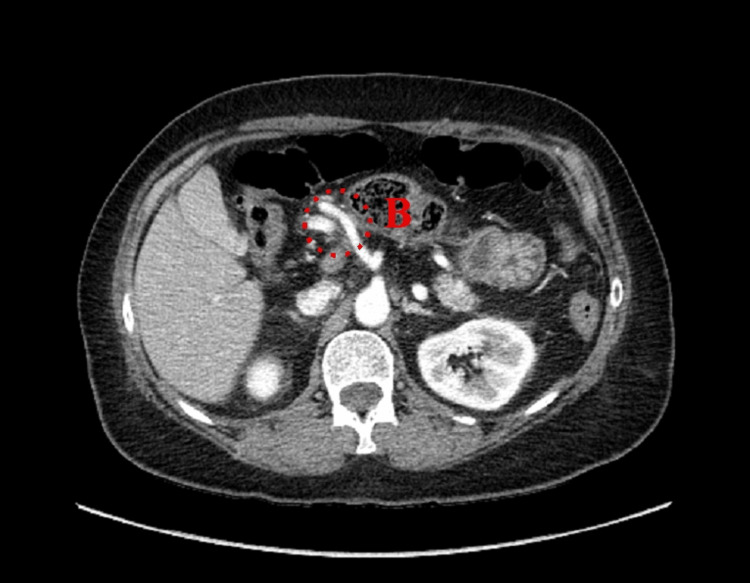
Axial view of Patient #3 (B) Colon inside FWH. Dashed circle: colon traveling through FWH. FWH: foramen of Winslow

Patient #3 is a 66-year-old female with a past medical history of a repaired hiatal hernia, gastroesophageal reflux disorder, hyperlipidemia, hypertension, and Raynaud’s syndrome. She presented to the emergency room with progressive epigastric pain, nausea, vomiting, and diarrhea. Initial differential diagnosis included gastritis, biliary colic, pancreatitis, cholecystitis, and cholelithiasis. An abdominal CT scan revealed an epiploic/FWH internal hernia, containing the cecum and terminal ileum, with increasing edema surrounding the mesenteric vessels and celiac axis (Figures 8-9). Additionally, the cecum was situated in the lesser sac and gastrohepatic ligament and was stool-filled, concerning for partial obstruction.

Similar to Patient #2, this patient had some resolution of pain after presentation to the hospital. Given ongoing imaging findings, surgical versus nonsurgical management options were discussed, and the patient agreed to proceed with laparoscopic robotic repair.

**Figure 10:**
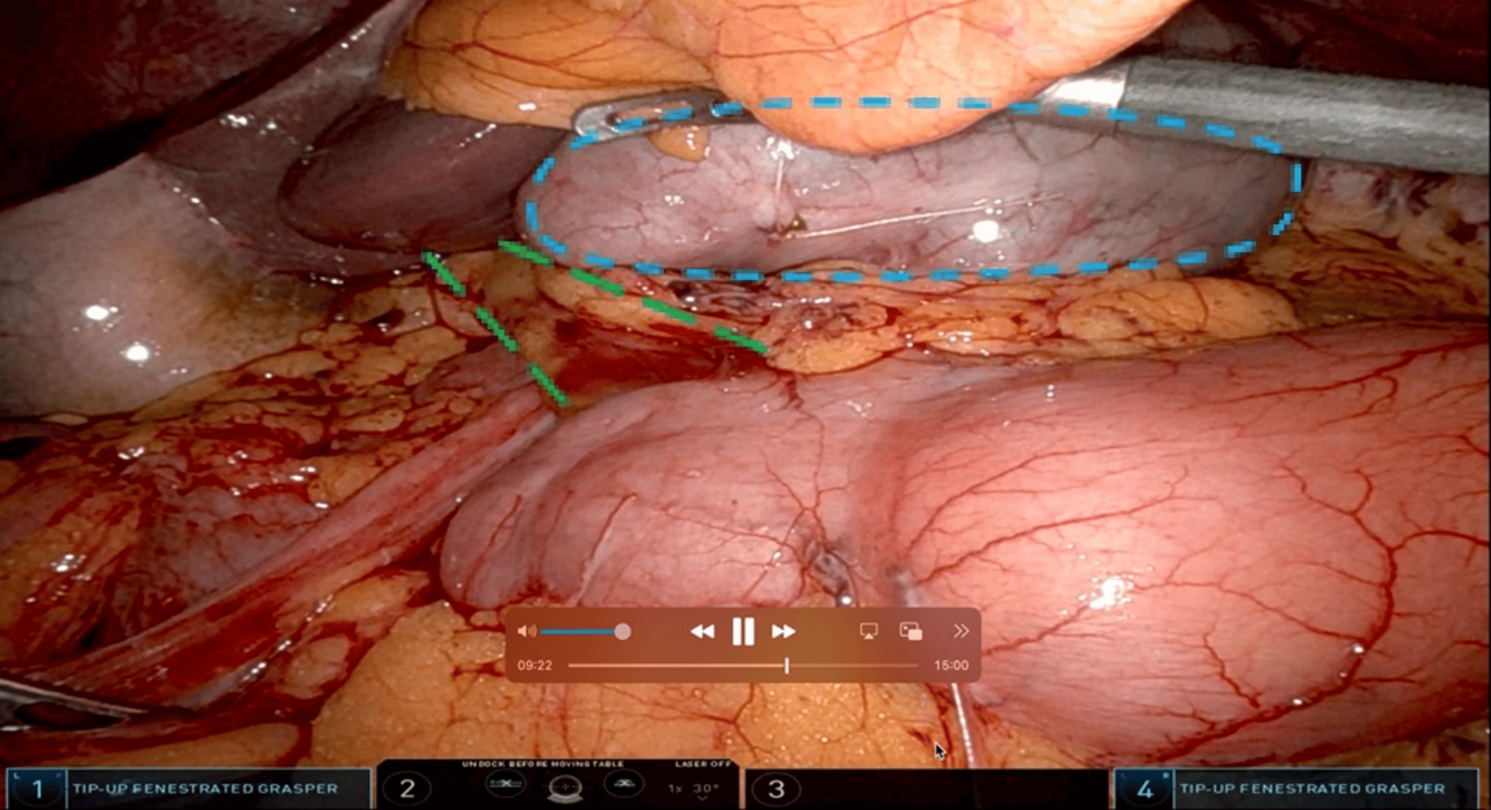
Laparoscopic view of Patient #3 prior to hernia reduction Cecum seen traveling through FWH. Dashed circle (blue): cecum. Dashed lines (green): hepatoduodenal ligament. FWH: foramen of Winslow

**Figure 11:**
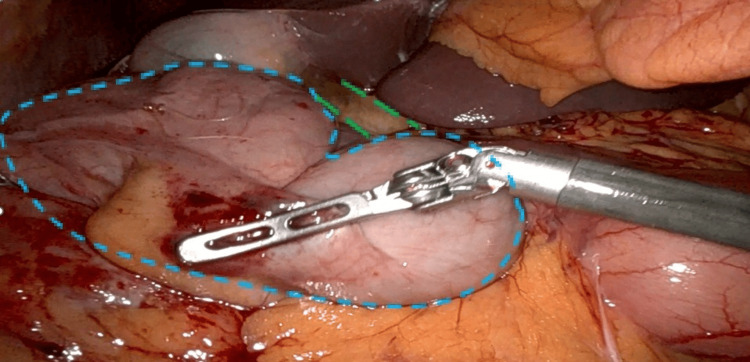
Laparoscopic view of Patient #3 after hernia reduction Cecum removed from FWH. Dashed circle (blue): cecum. Dashed lines (green): hepatoduodenal ligament. FWH: foramen of Winslow

An 8 mm robotic port with a 5-0 scope was used for abdominal entry at the left of the abdomen along the left anterior axillary line, at the level of the umbilicus. Three additional ports were placed at the same level as the left midclavicular line, the right midclavicular line, and the right anterior axillary line. Upon abdominal entry, it was immediately evident that the cecum had traversed through the FWH defect. Careful attempts at reduction were unsuccessful secondary to the high degree of gaseous distension. A pursestring suture was applied to the lateral aspect of the cecum and then punctured using a syringe and needle. Gas was aspirated from the cecum. Upon sufficient decompression, the pursestring was tied. This allowed for the reduction of the incarcerated cecum and small bowel through the defect (Figures 10-11). Upon complete reduction, a partial cecostomy was performed, excising the area of the pursestring with a 60 mm Echelon stapler port.

Next, an omental tongue of the transverse colon was fashioned with the Vessel Sealer. This flap was passed through the FWH defect. It was then secured with 3-0 STRATAFIX in a simple running fashion, anchoring it medially and laterally to both the lesser curvature of the stomach and the edge of the hepatoduodenal ligament. The right colon and cecum were subsequently fixed (cecopexy) to the lateral abdominal wall with 3-0 STRATAFIX in a simple running fashion. These maneuvers effectively limited colonic mobility to reduce the risk of recurrence. Patient #3 made a full recovery with no postoperative complications or recurrence at six months of follow-up.

In summary, the patient underwent a successful robotic reduction of the herniated cecum and ileum, partial cecectomy, placement of omental tongue in the foramen, and cecopexy.

Postoperative care and follow-up

Postoperative management included gradual dietary advancement, early ambulation, and deep vein thrombosis prophylaxis. There were no postoperative complications or recurrent symptoms at follow-up, ranging from two weeks to six months. A summary of cases is found in Table 1. This case series has been reported in line with the PROCESS Guideline [11].

**Table 1:** Overview of patient presentations and outcomes CT: computed tomography, FWH: foramen of Winslow

	Age/sex	Relevant history	Clinical presentation	Diagnostic findings	Surgical approach	Operations	Outcome
Case 1	62/female	Laparoscopic cholecystectomy, congenital malrotation	Epigastric pain, nausea	CT: cecal volvulus	Open	Hernia reduction, right hemicolectomy, placement of omental tongue in foramen	No recurrence
Case 2	51/female	Elongated right lobe of the liver	Epigastric pain	CT: internal lesser sac hernia	Robotic	FWH repair, placement of omental tongue in the foramen	No recurrence
Case 3	66/female	Hiatal hernia repair	Epigastric pain, nausea, vomiting, diarrhea	CT: FWH, partial large bowel obstruction	Robotic	Hernia reduction, partial cecectomy, placement of omental tongue in the foramen, cecopexy	No recurrence

## Discussion

FWH is the only natural connection between the greater and lesser peritoneal cavities, existing posterior to the hepatoduodenal ligament. Its borders are defined superiorly by the caudate lobe of the liver, inferiorly by the duodenum, and posteriorly by the inferior vena cava. Pressure from the surrounding organs, such as the duodenum, stomach, and liver, as well as nearby peritoneal attachments and the relatively small nature of the foramen, create an effective physiologic system to prevent herniation [12].

FWH remains rare, with fewer than 300 reported cases. The variety of clinical presentations and lack of a gold standard for diagnosis and treatment make both identifying and treating this pathology difficult. This case series describes three incidences of FWH with varying presentations and risk factors, all successfully treated operatively with no complications or recurrence.

Known risk factors include a hypermobile right colon, congenital malrotation, increased intra-abdominal pressure, large uterine fibroids, an elongated right liver lobe, and prior cholecystectomy [4,13]. Our series reflected these, with intraoperative findings of congenital malrotation and prior cholecystectomy (Case 1), hypermobile right colon and elongated liver lobe (Case 2), and hypermobile right colon and prior hiatal hernia repair (Case 3).

Clinical presentations of FWH typically occur as bowel obstructions, including acute epigastric pain, nausea, and vomiting. Rarely, patients may present with symptoms of obstructive jaundice if there is compression of the hepatic pedicle [8]. Each of our cases had a range of presenting symptoms with a commonality of epigastric pain with or without nausea and vomiting. Of note, two patients experienced at least partial resolution of their symptoms yet opted for operative management to prevent recurrent symptoms. The clinical presentation may be nonspecific, with a broad range of differential diagnoses, and FWH is often not considered due to its rarity. Cross-sectional imaging is a crucial tool in the preoperative diagnosis of internal hernia, where strangulation can result in mortality rates as high as 49% [14]. CT is the most efficient and timely imaging modality for the diagnosis of FWH. One of the most specific CT findings in FWH is the “bird’s beak sign,” characterized by the tapered herniated contents in the lesser sac pointing to the FWH, appearing as a beak [15,16]. Herniated contents include small bowel in 63% of cases, cecum and ascending colon in 30%, and transverse colon in the remaining 7% [15]. MRI may also be used for better characterization of these hernias; however, it may be less practical in emergent cases [17].

Though the majority of surgical treatments for FWH describe an open approach, the use of minimally invasive approaches is increasing [4]. Laparotomy remains important in unstable patients or those with failed laparoscopic reduction. Laparoscopy may offer improved visualization, as well as shorter hospital stay, reduced postoperative pain, and reduced risk of serious complications [18]. Ultimately, the approach should be chosen based on the surgeon's expertise and patient-specific factors [19].

An important consideration in the repair of FWH is how to manage the foramen defect and the hypermobile right colon after reduction. Described surgical techniques for this include closure of the foramen with primary suture repair or an omental flap, or enlarging the foramen to prevent future strangulation in case of recurrence. Alternatively, some choose not to close the foramen and instead perform a right hemicolectomy or cecopexy to prevent recurrence. If ischemia is present, a right hemicolectomy or ileocecectomy should be performed depending on the extent of ischemia. In cases of a hypermobile colon where the bowel remains viable, a cecopexy should be considered [20,21]. No instances of FWH recurrence have been reported, making it difficult to determine which management option is superior. Repair of FWH may complicate subsequent minimally invasive abdominal surgeries, such as cholecystectomy, given the alteration in surrounding anatomy and intra-abdominal adhesions. While there are no documented cases of severe complications in surgeries following FWH repair, it can be avoided with careful preoperative planning, as with any patient with a history of prior abdominal surgery.

## Conclusions

This case series details three rare cases of FWH that were treated surgically without complication or known recurrence, further contributing to the limited literature on this condition. Given its rarity, vague presentation, and inconclusive imaging, FWH should be considered in patients presenting with abdominal pain and signs of intestinal obstruction in the presence of known risk factors, such as prior cholecystectomy, congenital malrotation, or a mobile right colon. Surgical approaches should be chosen at the discretion of the operating surgeon based on their expertise; however, a minimally invasive approach is preferable when feasible, given lower complication rates and favorable postoperative recovery. Overall, further studies are needed to establish a gold standard for the surgical management of FWH.

## References

[1] Garg S, Flumeri-Perez G, Perveen S, DeNoto G (2016). Laparoscopic repair of foramen of Winslow Hernia. Int J Angiol.

[2] Deitrick J, Sessions W, Nguyen D, Santos A (2021). Cecal herniation through the foramen of Winslow: case presentation and literature review. J Clin Images Med Case Rep.

[3] Mouhafid FE, Njoumi N, Najih M (2018). A rare type of internal hernia: a case report and literature review. Int J Adv Res.

[4] Moris D, Tsilimigras DI, Yerokun B (2019). Foramen of Winslow hernia: a review of the literature highlighting the role of laparoscopy. J Gastrointest Surg.

[5] Winslow J-Bn, Donaldson A, Elliot C (1772). An anatomical exposition of the structure of the human body.

[6] Downs P, Downes N, Zayshlyy E, Esper C, Giuseppucci P (2018). Internal hernia through the foramen of Winslow. J Surg Case Rep.

[7] Huang Y, Qin L, Wu L, Huang Q (2022). An adolescent with ileum herniation through foramen of winslow: a case report and literature review. Niger J Clin Pract.

[8] Sikiminywa-Kambale P, Anaye A, Roulet D, Pezzetta E (2014). Internal hernia through the foramen of Winslow: a diagnosis to consider in moderate epigastric pain. J Surg Case Rep.

[9] Karlsen EA, Keogh C, Sandstrom A, Won CK (2021). Missed diagnosis of a foramen of Winslow internal hernia. ANZ J Surg.

[10] Leung E, Bramhall S, Kumar P, Mourad M, Ahmed A (2016). Internal herniation through foramen of Winslow: a diagnosis not to be missed. Clin Med Insights Gastroenterol.

[11] Agha RA, Mathew G, Rashid R (2025). Revised preferred reporting of case series in surgery (process) guideline: an update for the age of artificial intelligence. Prem J Science.

[12] Martin LC, Merkle EM, Thompson WM (2006). Review of internal hernias: radiographic and clinical findings. AJR Am J Roentgenol.

[13] Honma S, Itohara T, Sha S, Onoyama H (2021). Laparoscopic surgery in a patient with foramen of Winslow hernia due to large uterine fibroids: a case report and literature review. Surg Case Rep.

[14] Osvaldt AB, Mossmann DF, Bersch VP, Rohde L (2008). Intestinal obstruction caused by a foramen of Winslow hernia. Am J Surg.

[15] Lanzetta MM, Masserelli A, Addeo G (2019). Internal hernias: a difficult diagnostic challenge. Review of CT signs and clinical findings. Acta Biomed.

[16] Chen F, Bhatt S (2021). The beak sign of foramen of Winslow hernia. Abdom Radiol (NY).

[17] Elmohr MM, Blair KJ, Menias CO, Nada A, Shaaban AM, Sandrasegaran K, Elsayes KM (2020). The lesser sac and foramen of Winslow: anatomy, embryology, and CT appearance of pathologic processes. AJR Am J Roentgenol.

[18] Harnsberger CR, McLemore EC, Broderick RC (2015). Foramen of Winslow hernia: a minimally invasive approach. Surg Endosc.

[19] Almiron da R Soares G, de Oliveira Filho JR, Bregion PB (2025). Robotic vs laparoscopic vs open ventral hernia repair: insights from a network meta-analysis of randomized clinical trials. J Am Coll Surg.

[20] Buisset C, Postillon A, Aziz S, Bilbault F, Hoch G, Nesseler JP, Johann M (2020). Laparoscopic management of an ascending colon hernia through the foramen of Winslow. J Surg Case Rep.

[21] Luciano E, Hyde R, Solh W, Davis RT, Pacheco F (2021). Internal herniation of the cecum through the foramen of Winslow-a case report. J Surg Case Rep.

